# Transcriptome analysis of root response to citrus blight based on the newly assembled Swingle citrumelo draft genome

**DOI:** 10.1186/s12864-016-2779-y

**Published:** 2016-07-08

**Authors:** Yunzeng Zhang, Gary Barthe, Jude W. Grosser, Nian Wang

**Affiliations:** Citrus Research and Education Center, Department of Microbiology and Cell Science, IFAS, University of Florida, Lake Alfred, FL USA; Citrus Research and Education Center, Department of Horticultural Sciences, University of Florida, Lake Alfred, FL USA

**Keywords:** Citrus blight, Swingle citrumelo, Root, Genome assembly, RNA-seq

## Abstract

**Background:**

Citrus blight is a citrus tree overall decline disease and causes serious losses in the citrus industry worldwide. Although it was described more than one hundred years ago, its causal agent remains unknown and its pathophysiology is not well determined, which hampers our understanding of the disease and design of suitable disease management.

**Results:**

In this study, we sequenced and assembled the draft genome for Swingle citrumelo, one important citrus rootstock. The draft genome is approximately 280 Mb, which covers 74 % of the estimated Swingle citrumelo genome and the average coverage is around 15X. The draft genome of Swingle citrumelo enabled us to conduct transcriptome analysis of roots of blight and healthy Swingle citrumelo using RNA-seq. The RNA-seq was reliable as evidenced by the high consistence of RNA-seq analysis and quantitative reverse transcription PCR results (R^2^ = 0.966). Comparison of the gene expression profiles between blight and healthy root samples revealed the molecular mechanism underneath the characteristic blight phenotypes including decline, starch accumulation, and drought stress. The JA and ET biosynthesis and signaling pathways showed decreased transcript abundance, whereas SA-mediated defense-related genes showed increased transcript abundance in blight trees, suggesting unclassified biotrophic pathogen was involved in this disease.

**Conclusions:**

Overall, the Swingle citrumelo draft genome generated in this study will advance our understanding of plant biology and contribute to the citrus breeding. Transcriptome analysis of blight and healthy trees deepened our understanding of the pathophysiology of citrus blight.

**Electronic supplementary material:**

The online version of this article (doi:10.1186/s12864-016-2779-y) contains supplementary material, which is available to authorized users.

## Background

Citrus blight, a citrus tree overall decline disease caused by unknown agent, was first described over 100 years ago [1]. This disease normally occurs in hot and humid citrus-producing areas, including North America, the Caribbean, South America, South Africa and Australia. The typical blight symptoms include zinc deficiency in the leaves, twig die back and overall tree decline [2–5]. Expression of p12 and blockage of xylem tissues are always associated with blight trees [2–5]. Consequently, antibody against p12 and reduced water uptake into the trunk due to the xylem blockage are widely used for diagnosis of citrus blight [6, 7]. The causal agent of citrus blight remains unknown. The symptoms and characteristics associated with citrus blight can be reproduced by root graft inoculations but not by grafting canopy branches or by soil replacement, suggesting that citrus blight is caused by a systemic infectious agent, and the causal agent is restricted to the roots, as reviewed by Derrick and Timmer [5]. However, several observations found the disease spreading behavior fits a linear model, similar to abiotic abnormalities [5]. Multiple plant pathogens, such as *Xylella fastidiosa* [8], *Fusarium solani* [9, 10] and idaeovirus [11], as well as some abiotic factors, such as nitrogen nutrients [12], were hypothesized as causal agents of citrus blight, but none have been confirmed [5].

Plants have integrative signaling and response networks to adapt themselves to the ever-changing environments. For example, when facing drought stress, metabolism is reprogrammed, and the synthesis and signaling pathways of ABA are activated [13, 14]. When attacked by pathogens, plant immune responses are triggered, leading to dramatic changes in host transcriptional responses. Distinct features of host responses have been reported in response to infection by different pathogens or abiotic stresses [15, 16]. Therefore, investigation of the host transcription response will deepen our understanding of the pathophysiology and etiology of citrus blight. In a previous study, cDNA subtractive hybridization was used to identify differentially expressed genes in the roots of healthy and blighted rough lemon (*Citrus jambhiri* Lush) rootstock supporting sweet orange (*C. sinensis*). However, genome sequence was unavailable for citrus when this study was performed in 2004. Thus, little information was gained in this previous study [17]. Recently, the genomes of sweet orange (*C. sinensis*) and clementine mandarin (*C. clementina*) have been sequenced and assembled into draft genomes [18, 19], which facilitate the assembly and annotation of newly sequenced closely related citrus species. In addition, RNA-seq-based next-generation sequencing allows unprecedented opportunities to identify novel transcripts [20]. Thus, we aimed to revisit the citrus blight with the aid of the citrus genome sequences and next generation sequencing technologies. The draft genome of Swingle citrumelo, a very important rootstock in Florida [21], was sequenced, assembled and used as reference for RNA-seq analysis. More than 4000 differential expressed genes were identified using Tophat-Cufflinks-Cuffdiff pipeline [22], and their association with the citrus blight disease development and adaption was explored. Understanding the pathophysiology of citrus blight will provide hints for novel disease management and disease-resistant breeding improvement.

## Results

### Overview of the Swingle citrumelo draft assembly and annotation

The rootstock used in this study, Swingle citrumelo, is a hybrid from *Citrus paradisi* Macf. X *Poncirus trifoliata* (L.) Raf. (synonym: *Citrus* × *paradisi* × *Citrus trifoliate*, NCBI taxonomy ID: *309804*) [23]. It formed a separate phylogenetic clade from the two published citrus genomes (see Additional file 1: note 4 and Figure S1). To obtain a reliable reference genome for the RNA-seq analysis, the Swingle citrumelo draft genome was assembled based on a reference-assisted approach.

A total of 69,656,379 × 2 trimmed high quality pair-end DNA reads (13.6 Gigabases (Gb)), from sample 23_11 were subjected for assembling using CLC genomic workbench v6.0.1 (CLC Bio). The assembly produced by word size 33 contained 720.2 K contigs with average contig length 669 bp with a total length of 482 Mb. After removing low credential contigs (average coverage lower than 6) and non-citrus originated contigs, 136,559 scaffolds affiliated with citrus were combined together, scaffolded and gap filled as mentioned in supplementary files. The resulting assembly length was 280.6 Mb, which covered about 74 % of the estimated swingle genome (380 Mb per 1C genome) (0.788 pg/2C) [24]. The average coverage was approximately 15X. The assembly has been deposited in NCBI under accession no. AZHM00000000. The detailed assembly information was listed in Table 1.

**Table 1:** Overview of the draft assembly of Swingle citrumelo

Estimated genome size (Mb)	380
Genome assembly length (Mb)	280.6
Estimated coverage (%)	74
Number of scaffolds (≥500 bp)	66,319
Largest scaffold (kb)	234
N50 length (kb)	11.4
GC content (%)	34.44
N's length (Mb)	14
Repetitive element length (Mb)	44.8
Gene number	29,054

In total, 446 (97.4 %) of the 458 Core eukaryotic genes (CEGs) were identified in the assembly, and 97.8 % and 96.3 % of the 7954 swingle ESTs were aligned to the assembly using sim4db and exonerate, respectively, suggesting the draft assembly has a high coverage of coding sequences.

A total of 44.8 Mb (16.8 % of 280.6 Mb) of repetitive elements were identified in the draft assembly using RepeatMasker, generating a 235.8 Mb repeat-masked assembly for gene prediction. Following two cycles of MAKER run, 29,054 genes were predicted without detection of alternative splicing forms (see Additional file 1: note 3). This version of annotation was used as reference for RNA-seq analysis.

### Overview of the RNA-seq data

A total of 725 million high-quality, paired-end reads (approximately 91.8 % of the total raw reads) were generated from the seven root samples from healthy and blight trees after trimming using CLC genomic workbench V6.0.1 (CLC Bio), with 100.8 to 117.6 million reads from each sample. A total of 505.69 million reads (69.8 % of trimmed reads) could be mapped uniquely to one location within the draft assembly, whereas an additional 6.69 million reads (0.9 %) were mapped to multiple locations within the draft assembly (Table 2).

**Table 2:** Overview of mapped RNA-seq reads using Tophat2

Sample	Trimmed reads (million)	Unique mapping reads (million)	Multi-mapping reads (million)	Percentage of mapped reads (%)
14_14	117.6	82.61	1.13	71.6
16_11	100.8	70.51	0.97	70.9
20_2	91	60.85	0.77	67.7
18_7	98.8	70.31	0.90	72.1
20_6	105	72.12	1.00	69.6
23_11	107.8	76.01	1.01	71.4
24_8	104.6	73.28	0.91	70.9

Note: the data was calculated by RSeQC ver2.3.6 [62]

### Transcriptome analysis of roots of healthy and blight trees

The variation between the seven samples was assessed using a multidimensional scaling (MDS) plot based on the overall gene expression profiles prior to differential expression analysis. In the MDS plot, the two healthy trees (20_6 and 24_8) formed a group, the two blight trees (14_14 and 16_11) clustered closely, and the two pre-blight trees (18_7 and 23_11) and the late blight stage tree (20_2) stood alone and were separated from the other trees (Additional file 1: Figure S2). Specifically, the healthy trees remained healthy in a two-year duration, whereas the pre-blight trees showed no obvious blight symptoms at the beginning, but were diagnosed as blight later, *Candidatus* Liberibacter asiaticus, the causal agent of Huanglongbing was also detected in sample 18_7 but not in other samples.

Plant gene expression can be affected by many factors. The seven samples were collected from the same citrus grove under the same agricultural practices to minimize influence of environmental factors on the differential gene expression between blight and healthy trees. In addition, to rule out the possibility of differential gene expression caused by genetic difference, a phylogenetic tree was constructed using single nucleotide polymorphism (SNP) of the seven samples. SNPs were called by mapping DNA reads from all seven samples to the Swingle citrumelo assembly. The phylogenetic tree revealed the seven samples were nearly identical (Additional file 1: Figure S1). Among the first 10,000 SNP sites, 9995 sites were identical for all seven samples, further demonstrating the samples used were from nucellar seedlings. Thus the possibility of genomic background difference caused by zygotic seedlings was ruled out.

To investigate the differential gene expression between blight and healthy samples, we focused on the two healthy trees (20_6 and 24_8) and two blight trees (14_14 and 16_11), which had more consistent intragroup expression profiles. The two pre-blight samples were not closely grouped, thus eliminated from further analysis due to lack of replication (Additional file 1: Figure S2C). Using a stringent cutoff: 2-fold change, q-value ≤ 0.05 and FPKM ≥1, 4440 differentially expressed genes (DEGs) including 2383 down-regulated genes and 2057 up-regulated genes (blight vs. healthy), were identified (Additional file 2).

### The metabolism pathways overview

**Fig. 1:**
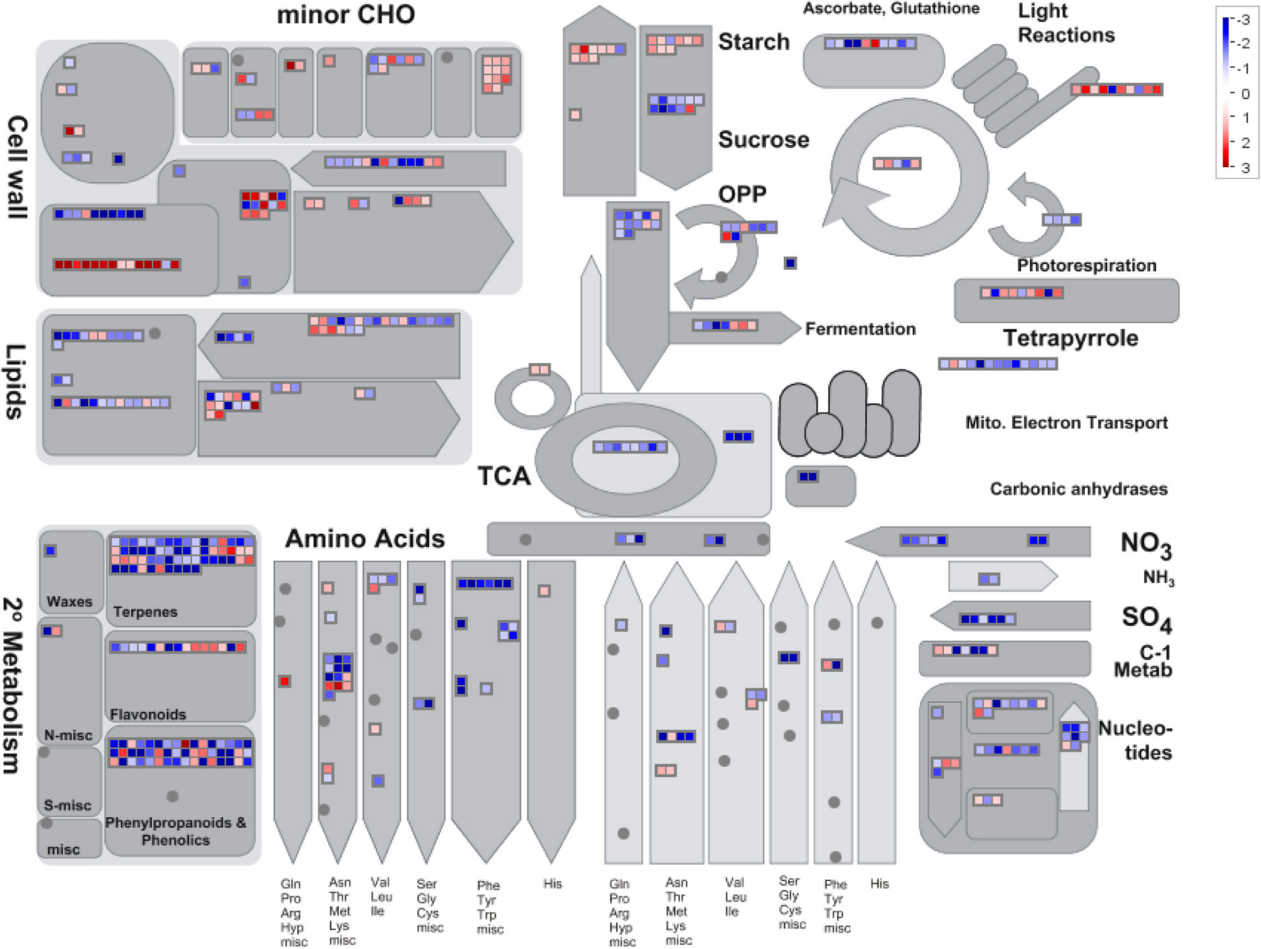
Differentially expressed genes associated with metabolic processes (blight vs. healthy). The figure was generated using MapMan software. Blue denotes down-regulated genes, and red denotes up-regulated genes. The log2-fold change in the transcript levels was used for the analysis

In the down-regulated genes in blight trees, metabolism related GO SLIM terms were enriched, including “biosynthetic process, GO:0009058”, “catabolic process, GO:0009056”, “lipid metabolic process, GO:0006629”, “carbohydrate metabolic process, GO:0005975”, “secondary metabolic process, GO:0019748” and “cellular protein modification process, GO:0006464” (Table 3). Further analysis using MapMan indicated that the DEGs involved in the TCA cycle, mitochondrial electron transport, and nitrogen assimilation, and most of the DEGs involved in amino acid metabolism, lipid metabolism, glycolysis, secondary metabolism and nucleotide metabolism, were down-regulated in blight trees (Fig. 1). The down-regulation of metabolism genes is consistent with the decline of blight trees.

**Table 3:** The enriched GO terms and plant GO SLIM terms in the differentially expressed genes (DEGs) in the blight trees compared to healthy tree revealed by Blast2GO

GO-ID	Term	FDR	P-Value	#Test	#Ref	#not annot in Test	#not annot in Ref
Down-regulated plant GO SLIM terms							
GO:0009058	Biosynthetic process	5.12E-05	1.58E-06	660	5048	1241	12083
GO:0019748	Secondary metabolic process	1.94E-04	7.39E-06	235	1569	1666	15562
GO:0009056	Catabolic process	4.05E-04	2.11E-05	364	2643	1537	14488
GO:0006629	Lipid metabolic process	0.015215	0.001265	264	1964	1637	15167
GO:0006464	Cellular protein modification process	0.017532	0.001541	271	2030	1630	15101
GO:0005975	Carbohydrate metabolic process	0.027641	0.002561	327	2522	1574	14609
Up-regulated GO terms							
GO:0006075	(1- > 3)-beta-D-glucan biosynthetic process	0.013333	3.99E-05	11	22	1555	17444
GO:0080165	Callose deposition in phloem sieve plate	0.034774	1.48E-04	7	9	1559	17457
GO:0005982	Starch metabolic process	0.034774	1.54E-04	73	505	1493	16961

Note: #Test: the number of DEGs in the listed gene set; #Ref: the number of genes belonging to the listed gene set annotated in the Swingle citrumelo genome; # not annot in Test: the number of DEGs not belonging to the listed gene set; # not annot in Ref: the number of genes not belonging to the listed gene set annotated in the Swingle citrumelo genome

**Fig. 2:**
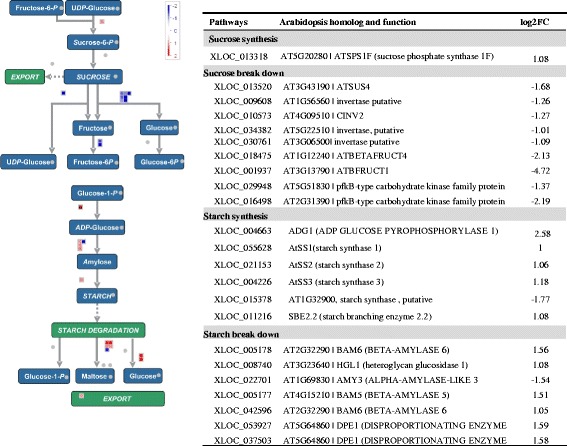
Differentially expressed genes associated with starch and sucrose metabolism (blight vs. healthy). The figure was generated using MapMan software. Blue denotes down-regulated genes, and red denotes up-regulated genes (Blight vs. Healthy). The log2-fold change in the transcript levels was used for the analysis

Although most of the metabolic pathways showed reduced transcription activity in blight trees, the GO term “starch metabolic process, GO:0005982” was enriched with up-regulated genes under blight conditions (Table 3). Furthermore, the MapMan analysis demonstrated that a small subunit of ADP-glucose pyrophosphorylase (XLOC_004663), which catalyzes the first step of starch synthesis, three starch synthases (XLOC_055628, XLOC_021153, XLOC_004226) and a starch branching enzyme (XLOC_011216) were up-regulated in blight trees. Numerous genes encoding enzymes involved in starch degradation, including starch cleavage and disproportionation, were also up-regulated in blight trees. A gene encoding sucrose phosphate synthase, responsible for sucrose synthesis, was up-regulated, whereas multiple genes involved in sucrose degradation pathways were down-regulated in blight trees (Fig. 2).

### The hormone related genes

Significant alteration in expression for genes involved in hormone related pathways was observed between the blight and healthy samples. Two genes, XLOC_027196 and XLOC_027197, which were annotated as homologs of *Arabidopsis* aldehyde oxidase 1 (*AAO1*) and *AAO3*, respectively, were up-regulated in blight trees. The *AAO3* gene encodes an enzyme that catalyzes the final step of ABA biosynthesis and plays a crucial role in ABA synthesis, whereas the AAO1 gene may contribute partially to ABA biosynthesis [25]. Two ABA3 genes (XLOC_011831 and XLOC_011832), which are the key regulator of ABA biosynthesis, were up-regulated. In addition, XLOC_028331 and XLOC_010730, which encode ABA responsive elements-binding factor 3 (ABF3) and ABF4 respectively, were also induced in blight trees (Additional file 1: Table S1).

**Fig. 3:**
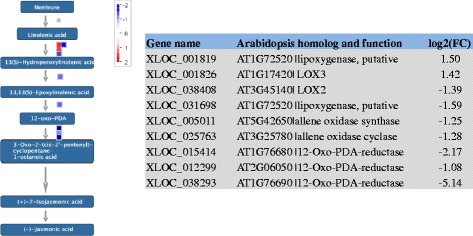
The JA synthesis pathway was repressed in blight trees. Blue denotes down-regulated genes, and red denotes up-regulated genes (Blight vs. Healthy). The figure was generated using MapMan software. The log2-fold change in the transcript levels was used for the analysis

Several genes involved in JA synthesis, such as allene oxidase synthase, allene oxidase cyclase and 12-Oxo-PDA-reductase genes, were down-regulated in blight trees (Fig. 3). Down-regulation of the ethylene synthesis genes 1-aminocyclopropane-1-carboxylate oxidase (ACO) gene ACO1 (XLOC_012043) and ACO4 (XLOC_004937) was observed in blight trees. Furthermore, the ethylene responsive factors (ERFs), including ERF1, the crucial defense signaling factor for JA and ethylene signaling pathway, were down-regulated in blight trees (Additional file 1: Table S1).

### The plant defense system

Two GO terms, (1- > 3)-beta-D-glucan biosynthetic process (GO:0006075) and callose deposition in phloem sieve plate (GO: 0080165), with 11 genes included, were enriched in the blight up-regulated gene set (Table 3), and all the 11 genes were annotated as glucan synthase-like (Gsl) genes (also known as callose synthase (Cals) genes).

The mitogen-activated protein kinase gene MPK4 (XLOC_037129) and its upstream activator mitogen-activated protein kinase kinase (MKK) gene MKK2 [26] (XLOC_012257) were down-regulated in blight trees. MPK4 is known as a negative regulator of SA-dependent systemic acquired resistance as well as a positive regulator of ET and JA mediated defense [27, 28]. The down-regulation of MPK4 suggests that the JA and ET-related pathways were repressed, whereas the SA-related defense pathways were activated in blight trees. In fact, the JA and ET synthesis and signaling pathways were down-regulated (see above); in contrast, several SA-induced genes showed increased transcript abundance in blight trees. The NPR1 (nonexpresser of PR genes 1) (XLOC_012174, q-value = 0.038, log2FC = 0.93), which plays a key role in the SA-mediated defense signaling pathways, was up-regulated. NPR3 (XLOC_022440) and NPR4 (XLOC_022463 and XLOC_022469), which act as adaptor proteins for CUL3 E3 ligase to degrade NPR1 specifically [29], were down-regulated in blight trees. In addition, expression of the downstream pathogenesis-related (PR) gene PR1 (XLOC_026451) and MPK3 (XLOC_027731) was up-regulated in blight trees. Up-regulation of three SA-related WRKY transcription factors, WRKY33 (XLOC_019616), WRKY53 (XLOC_037606) and WRKY70 (XLOC_019719) and a gene encoding glutaredoxin (XLOC_004271) was also observed.

### RT-qPCR validation of differentially expressed genes from RNA-seq

**Fig. 4:**
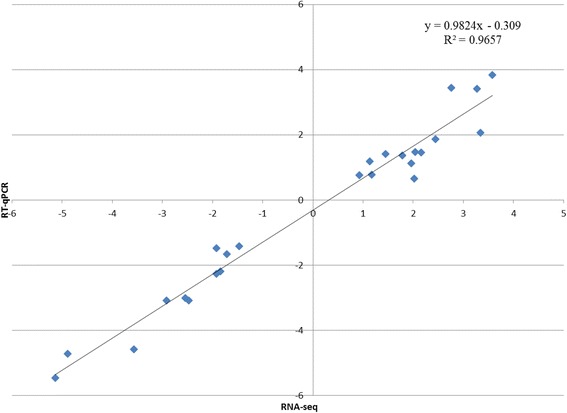
RT-qPCR validation of differentially expressed genes. The log2FC values from the RNA-seq results are displayed on the x-axis, and the values from the RT-qPCR are displayed on the y-axis. The high R^2^ (0.966) indicates the results from the RT-qPCR and RNA-seq are consistent

Twenty-five genes in the DEGs, including 11 down-regulated and 14 up-regulated genes, were selected for RT-qPCR assay to validate the RNA-seq results (Fig. 4 and Additional file 1: Table S2). As shown in Fig. 4, the RT-qPCR results suggested that the expression patterns of these genes were consistent with the RNA-seq data (R^2^ = 0.966).

## Discussion

Eight citrus cultivars have been sequenced, and two of them, sweet orange (*Citrus sinensis*) and *C. clementina,* were assembled to draft genomes [18, 19]. Swingle citrumelo was sequenced and assembled in this study. Swingle citrumelo is the most important rootstock in Florida, accounting for 37 % of all propagations as of 2012 [21]. Swingle citrumelo is a hybrid from *Citrus paradisi* Macf. X *Poncirus trifoliata* (L.) Raf. [23]. The phylogenetic tree, which was constructed using the SNPhylo pipeline [30], demonstrated that Swingle citrumelo formed a separate clade from the clade containing the two published citrus genomes (see Additional file 1: note 4 and Figure S1). Given the high variability of citrus cultivars, mainly due to hybridization during their complex evolutionary history [19], it was crucial to assemble the Swingle citrumelo genome to obtain a reliable annotation for RNA-seq analysis. The high consistence between RNA-seq and RT-qPCR results demonstrated the reliability of the assembly. The Swingle citrumelo draft genome will advance our understanding of plant biology and contribute to the citrus breeding.

Transcriptome analysis of blight and healthy citrus plants provides insights to the molecular mechanism to the characteristic blight phenotypes. Firstly, the GO enrichment analysis and MapMan metabolism overview results demonstrated that many metabolism pathways were down-regulated in the roots of blight trees. The reduced metabolism in the roots is consistent with the overall decline of blight trees since roots are critical for plants to absorb water and nutrition (e.g., nitrogen and mineral elements). Secondly, the expression pattern of starch related genes is in agreement with the observation of a large number of starch grains in the parenchyma cells of the phloem [31]. Furthermore, the gene expression pattern of blight trees is consistent with the drought stress due to xylem blockage [6]. Starch synthesis genes were up-regulated in blight trees (Fig. 2), leading to starch deposition. Accumulation of starch has been suggested to help plants adapt to drought stress [32]. ABA also plays a key role in adaptive responses to drought stress [14]. Drought-mediated, up-regulation of ABA synthesis and ABA-induced downstream genes are widely reported [13, 33, 34]. Numerous genes involved in ABA synthesis and signaling showed increased transcript abundance in blight trees including ABA3, AAO1, AAO3, ABF3 and ABF4. AAO3 plays a key role in catalyzing the last step of the ABA synthesis pathway to help plants adapt to drought stress, which has been determined in leaves and seeds [25, 35], and this gene is actively expressed in many tissues, including roots, stems and leaves [36]. Consistently, members of the ABF gene family function in ABA signaling and can be induced under abiotic stresses, including drought stress [13, 37–39].

Plant hormones play key roles in regulating immune responses to a wide range of pathogens. Among them, SA, JA and ET (ethylene) are the most prominent. SA signaling triggers resistance against biotrophic and hemibiotrophic pathogens; in contrast, JA and ET work together to play an important role in defense against necrotrophs [40, 41]. SA-mediated and JA- and/or ET-dependent defense pathways antagonize each other, and cooperation between them is not commonly observed [15, 42]. Our RNA-seq results suggested the JA and ET biosynthesis pathways including ERF1 and ERF2 [43, 44], were down-regulated in blight trees (Fig. 3). ERF proteins are responsible for regulating the expression of JA- and ET-mediated disease resistance response genes by binding to the GCC box (AGCCGCC) in their promoter regions [45]. Two known important positive regulators of JA- and ET- dependent, defense-related transcription, were identified in the down-regulated genes, suggesting the JA and ET-mediated immune systems were repressed in blight trees. On the other hand, up-regulation of NPR1, the central regulator of SA-dependent gene activation and SAR [46], and down-regulation of NPR3 and NPR4, which work as adapters for NPR1 degradation [29], as well as the up-regulation of downstream defense genes (e.g., PR1 and MPK3), indicated that the SA-mediated defense system was activated. The down-regulation of MPK4, which acts as a negative regulator of SA signaling and a positive regulator of JA signaling [28], as well as the up-regulation of glutaredoxin gene, which can be induced by SA and suppresses JA-responsive *PDF1.2* transcription [47], further supported our notion. Furthermore, callose ((1,3)-β-glucan polymer) synthesis and deposition can play an important role in the defense response to invading pathogens [48, 49]. We observed the induction of 11 CalS genes in blight diseased trees (Table 3). In *Arabidopsis*, the expression of CalS1 (Gsl-6) and CalS2 (Gsl-3) is NPR1-dependent [50]. The induction of SA-dependent defense genes and the repression of JA- and ET-mediated immune systems supported the proposition that a systemic infectious agent, probably an unidentified biotrophic pathogen, is involved in citrus blight, as suggested by Derrick and Timmer [5]. However, given the fact that the plant hormone signaling pathways are well programmed by plant to respond to both biotic and abiotic stresses, the possibility of certain abiotic stress contributed to the observed antagonism between JA-ET dependent and SA mediated signaling pathways could not be ruled out [51].

## Conclusions

In conclusion, we have sequenced and assembled the draft genome of Swingle citrumelo. We also analyzed the root transcriptome of blight and healthy trees. The transcriptome analysis provides insights to the molecular mechanism underneath the characteristic blight phenotypes including decline, starch accumulation, and drought stress. The activation of SA-dependent defense pathways and the repression of JA- and ET- mediated defense pathways were observed in blight trees, further supporting that unidentified biotrophic pathogen(s) was/were involved in citrus blight. This study deepened our understanding of the pathophysiology of citrus blight. However, the causal agent remains undetermined and yet to be determined.

## Methods

### Plant materials, nucleic acid extraction and sequencing

Sweet orange on Swingle citrumelo [*Citrus paradise* Macf. ×*Poncirus trifoliata* (L.) Raf.] (synonym: *Citrus* × *paradisi* × *Citrus trifoliate*, NCBI taxonomy ID: 309804) rootstock in a citrus grove located at St. Cloud (28°15’ N, 81°14’ W) was selected to collect root samples from healthy and blight trees. These trees were approximately 20 years old. The trees were diagnosed as blight-diseased based on visual symptoms, the p12 immunoassay on leaf tissues (Additional file 1: Figure S2), and water uptake results [6, 7]. The trees were followed for two years for disease development after sampling. Based on initial and later diagnoses, the trees were classified as healthy (2 trees, 20_6 and 24_8, water uptake >20 ml/30 s in all tests during the two years period), pre-blight (2 trees, 18_7 and 23_11, healthy when collected samples but diagnosed as blight one year after the initial diagnosis, water uptake >20 ml/30 s for the initial survey but <5 ml/30 s for the second survey), blight (2 trees, 14_14 and 16_11, water uptake =0 ml/30 s for two years tests) and late-blight (1 tree, 20_2, the symptoms were very severe at one year after the initial diagnosis) (Additional file 1: Figure S2). Around 10 cm lateral root segments (diameter approximately 0.5 cm) were collected from four corners of one tree and were pooled together. Two root segments from two individual lateral roots were harvested from each corner and eight segments were collected for each tree. The rhizosphere soil was removed, and roots were cleaned by repeated washing (3 rinses) using pre-cooled ddH_2_O. Then, the roots were flash-frozen in liquid nitrogen. All the steps were performed on site. When back to laboratory, the frozen materials were stored at −80 °C until use. The woody part was removed with pre-cooled knife, and the remaining part of the roots was grounded to a homogenous powder using a sterilized mortar and pestle and liquid nitrogen.

Total RNA was extracted using the RNeasy plant mini kit, and contaminating genomic DNA was removed by performing the following On-column DNase digestion step, according to the manufacturer’s protocol (Qiagen). The integrity of the RNA was verified on an Agilent RNA6000 Pico Chip. Plant ribosomal RNA was depleted using the Ribo-Zero™ Magnetic kit (PNMRZSR116) according to the manufacturer’s instructions (Epicenter Technologies). The RNA-Seq libraries were prepared using the Script Seq v2 RNA-Seq library preparation kit (Epicenter Technologies). Total DNA was extracted using the DNeasy plant maxi kit (Qiagen). DNA libraries were prepared through a semi-automated procedure using a Beckman Coulter SPRI-TE™ workstation. The RNA and DNA samples were sequenced on an Illumina HiSeq 2000 platform for 101 cycles in both directions.

### Swingle citrumelo draft genome assembly and annotation

The 81,496,678 × 2 paired-end raw DNA reads from tree 23_11 were trimmed using CLC genomic workbench (V6.0.1, CLC Bio) with the following the parameters: minimum quality score 0.05, maximum number of ambiguities 2 and discarding the reads containing adapters or reads shorter than 55 bp. A total of 69,656,379 × 2 high quality paired-end reads with average length 97.6 bp were kept and were assembled using CLC genomic workbench (V6.0.1, CLC Bio) with an iterative adaptive assembly approach with a range of word sizes. The contigs generated by the assembly of word size 33 were chosen for further analysis because a word size of 33 produced the longest (on average) contigs and the highest matched reads. The citrus-originated contigs were extracted, scaffolded and gap-filled using a reference-based approach [18]. The completeness and accuracy of the coding region of the draft assembly were validated using the Core Eukaryotic Genes Mapping Approach (CEGMA) [52] as well as EST mapping using sim4db and exonerate [53, 54], respectively. The annotation of this draft genome was created using MAKER2 pipeline [55]. The detailed genome assembly and annotation pipeline are listed in the Additional file 1: notes 1–3.

### Differential expression analysis

The differential gene expression between healthy and blight citrus samples was analyzed following the tuxedo pipeline [22]. In brief, the RNA-seq reads were trimmed as described above. The trimmed reads were mapped to the Swingle citrumelo draft assembly using Tophat2 (ver. 2.0.7), and the generated alignments were fed to Cufflinks (ver. 2.1.1) for transcript assembly. The assemblies from individual samples were merged with the annotation set generated by MAKER2 using Cuffmerge. Gene differential expression analysis was performed using Cuffdiff2 [56], the two healthy trees (20_6 and 24_8) and two blight trees (14_14 and 16_11) were used in the analysis and group-wise comparison was conducted. The results were explored using CummeRbund (http://compbio.mit.edu/cummeRbund/). Only genes fitting the following cutoff: |fold change| ≥ 2, q-value ≤ 0.05 and FPKM ≥1, were considered as significantly differentially expressed genes (DEGs).

The GO terms and plant GO SLIM terms were assigned to the annotated genes of the Swingle citrumelo assembly, and the gene set enrichment analysis of the DEGs was conducted using Blast2GO pipeline [57]. The GO terms and the plant GO SLIM terms were assigned to transcripts based on the b2gsep13 GO database. The up-regulated and down-regulated DEG transcript lists were used as input for the one-tailed Fisher’s Exact Test in Blast2GO to identify the enriched GO terms. In addition, the MapMan gene functional categories (Bins) were assigned to DEGs using Mercator [58], and differentially regulated bins were identified using MapMan [59].

### RT-qPCR analysis

Total RNA from the same samples used for RNA-seq was reverse-transcribed to cDNA with oligo(dT)_20_primer using the SuperScript III First Strand Synthesis System (Invitrogen, Life Technologies). The gene expression patterns of FBOX, SAND, GAPC2 (GAPDH) and UPL7, which are recommended as superior reference genes by Mafra et al. [60], were checked from the Cuffdiff result, and GAPC2 was chosen as the reference gene because of its high and constant expression level in our samples. The primers for the target genes (Additional file 1: Table S3) were designed using the Primer3 (Ver. 0.4.0) online tool (http://bioinfo.ut.ee/primer3-0.4.0/). For each primer set, cDNA corresponding to 10 ng of total RNA was subjected to qPCR using a QuantiTect SYBR Green PCR Kit (Qiagen). The qPCR was performed on an Applied Biosystems 7500 fast Real-Time PCR System (Life Technologies). The PCR thermal cycling conditions were as follows: an initial step at 95 °C for 15 min and 40 cycles of 15 s at 95 °C, 30 s at 55 °C and 30 s at 72 °C. The signal was collected at the 72 °C step. The ΔΔC_t_ method [61] was used to analyze the results.

## Abbreviations

ABA, Abscisic acid; CB, citrus blight; ET, ethylene; FPKM, Fragments Per Kilobase of transcript per Million mapped reads; JA, Jasmonic acid; SA, Salicylic acid

## Additional files

Additional file 1: Supplementary figures 1 and 2, supplementary tables 1–4 and supplementary notes for genome assembly, annotation and phylogenetic tree construction. (Word, 1.2 Mb). Including **Figure S1.** The citrus phylogenetic tree constructed with SNP data from Swingle citrumelo (this study) and 8 citrus cultivars by SNPhylo (page 2); **Figure S2.** Analysis of citrus trees used for transcriptome analysis; **Table S1.** Differentially expressed genes related to ERFs and ABA pathways annotated by MapMan; **Table S2.** The qRT-PCR validation values for the 25 selected genes; **Table S3.** Primer sequences used to amplify the selected genes; **Table S4.** The NCBI accession no. for DNA and RNA reads as well as the draft assembly. (DOCX 1250 kb) Additional file 2: All the DEGs identified by cuffdiff2 when cutoff |FC| > =2, FDR < =0.05 and FPKM > =1 was applied. (XLSX 270 kb)
