# Supplementary material for: Transcriptome analysis of root response to citrus blight based on the newly assembled Swingle citrumelo draft genome
Source: BMC Genomics. 2016 Jul 8;17:485. doi: 10.1186/s12864-016-2779-y (PMC4938905; doi:10.1186/s12864-016-2779-y)
Supplement: Additional file 1: — Supplementary figures 1 and 2, supplementary tables 1–4 and supplementary notes for genome assembly, annotation and phylogenetic tree construction. (Word, 1.2 Mb). Including Figure S1. The citrus phylogenetic tree constructed with SNP data from Swingle citrumelo (this study) and 8 citrus cultivars by SNPhylo (page 2); Figure S2. Analysis of citrus trees used for transcriptome analysis; Table S1. Differentially expressed genes related to ERFs and ABA pathways annotated by MapMan; Table S2. The qRT-PCR validation values for the 25 selected genes; Table S3. Primer sequences used to amplify the selected genes; Table S4. The NCBI accession no. for DNA and RNA reads as well as the draft assembly. (DOCX 1250 kb) [file 12864_2016_2779_MOESM1_ESM.docx]

**Figure S1.** The citrus phylogenetic tree constructed with SNP data from Swingle citrumelo (this study) and 8 citrus cultivars [1] by SNPhylo [2]. The reads from each sample were aligned to the *Citrus sinensis* genome [3] using Bowtie2 (ver. 2.0.6) and SNPs were extracted using Samtools (ver. 0.1.19+). The SNPs were fed to SNPphylo to contrast the phylogenetic tree. 100 bootstrap replicates were made, and bootstrap values were indicated at each node. The genomes of *C. sinensis* and *C. clementina* have been published. The plot was generated using Figtree with a midpoint root. The scale bar indicates the average number of nucleotide substitution per site between the two nodes in the tree.

**Figure S2.** Analysis of citrus trees used for transcriptome and microbiome analyses. (A) and (B) p12 assay results from two independent replicates. (C) Multi-dimensional scaling (MDS) plot of gene expression of seven RNA-seq data. The figure was generated using cummerbund R package based on the cuffdiff2 output. B0 denotes sample 14_14; B1, 16_11; B2, 20_2; H0, 20_6, H1, 24_8; P0, 18_7; P1, 23_11. The photos were taken at the first sampling time and showed the visual symptoms of citrus blight.

**Figure S1.**

**
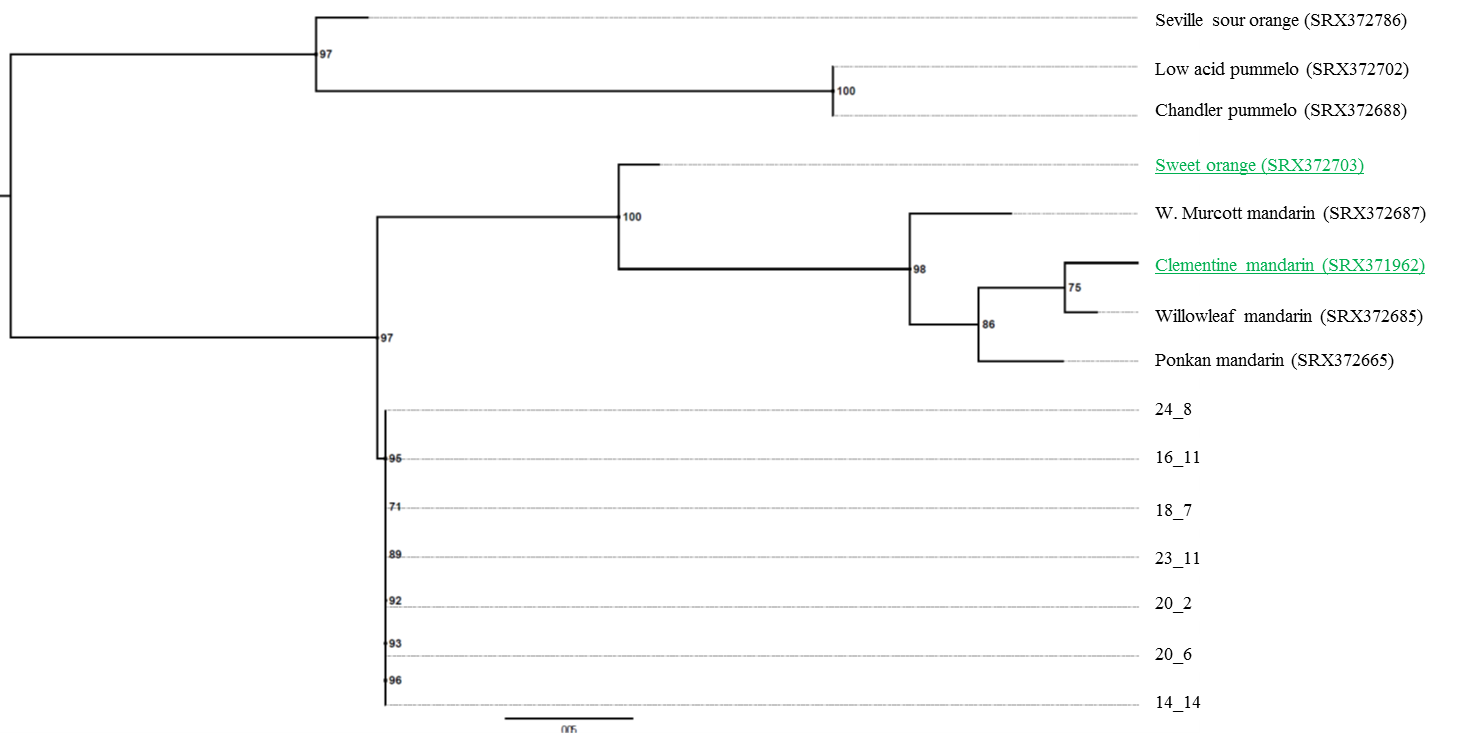
**

**Figure S2.**

**
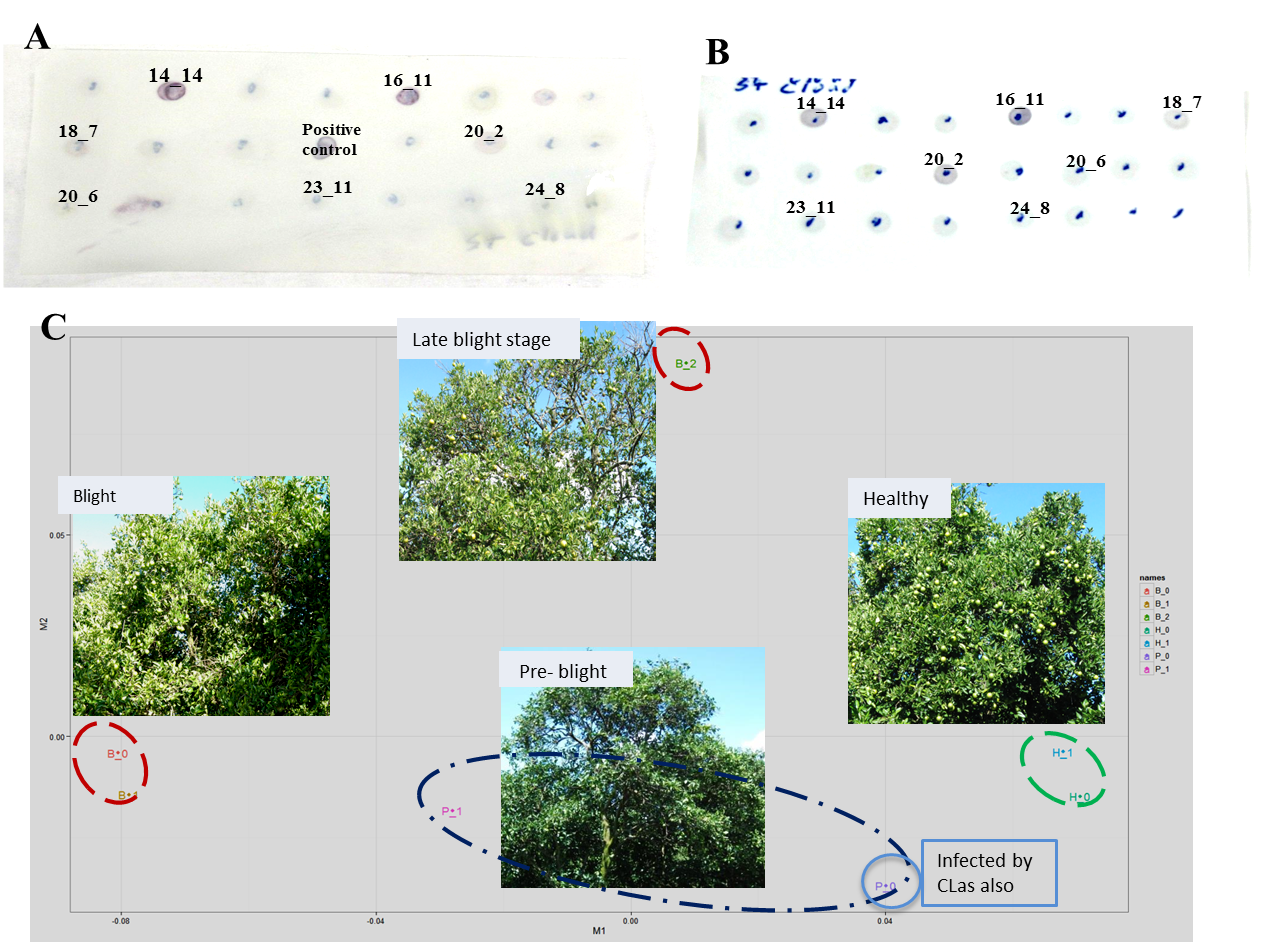
**

**Supplementary table 1.** Differentially expressed genes related to ERFs and ABA pathways annotated by MapMan

| MapMan  Bin | | gene number | AGI annotation by MapMan | log2(FC) | FDR |
| --- | --- | --- | --- | --- | --- |
| ERFs | | | | | |
| 27.3.3 | RNA.regulation of transcription.AP2/EREBP, APETALA2/Ethylene-responsive element binding protein family | XLOC_013553 | AT5G21960 \| Symbols: \| AP2 domain-containing transcription factor | 1.87693 | 0.028714 |
|  |  | XLOC_022621 | AT5G13330 \| Symbols: Rap2.6L | -2.56974 | 0.000411 |
|  |  | XLOC_023625 | AT5G25190 \| Symbols: \| ethylene-responsive element-binding protein | -1.18249 | 0.030985 |
|  |  | XLOC_001700 | AT1G53910 \| Symbols: RAP2.12 | -1.38304 | 0.000411 |
|  |  | XLOC_010186 | AT1G21910 \| Symbols: \| AP2 domain-containing transcription factor family protein | 1.99747 | 0.000411 |
|  |  | XLOC_017562 | AT3G23240 \| Symbols: ERF1 | -1.71518 | 0.000411 |
|  |  | XLOC_017561 | AT3G23230 \| Symbols: \| ethylene-responsive factor | -3.79432 | 0.000411 |
|  |  | XLOC_029507 | AT4G17500 \| Symbols: ATERF-1 | -1.76783 | 0.000411 |
|  |  | XLOC_029996 | AT5G52020 \| Symbols: \| AP2 domain-containing protein | -1.67727 | 0.000411 |
|  |  | XLOC_025759 | AT1G68840 \| Symbols: RAV2 | -1.87449 | 0.000411 |
|  |  | XLOC_010498 | AT5G50080 \| Symbols: ERF110 \| DNA binding / transcription factor | -4.0628 | 0.000411 |
|  |  | XLOC_029413 | AT3G16770 \| Symbols: RAP2.3 | -1.1288 | 0.000411 |
|  |  | XLOC_028321 | AT4G34410 \| Symbols: RRTF1 | 2.66748 | 0.000411 |
|  |  | XLOC_005687 | AT1G28360 \| Symbols: ERF12 | -1.05481 | 0.003853 |
|  |  | XLOC_002085 | AT2G44840 \| Symbols: ATERF13 | -3.1752 | 0.000411 |
|  |  | XLOC_005521 | AT5G47220 \| Symbols: ATERF2 | -2.33672 | 0.000411 |
|  |  | XLOC_013322 | AT1G51120 \| Symbols: \| AP2 domain-containing transcription factor | -2.41553 | 0.000411 |
|  |  | XLOC_033365 | AT2G20880 \| Symbols: \| AP2 domain-containing transcription factor | -1.62827 | 0.000411 |
|  |  | XLOC_027740 | AT2G28550 \| Symbols: RAP2.7 | -2.0264 | 0.000411 |
|  |  | XLOC_040420 | AT4G36920 \| Symbols: AP2 | -2.24381 | 0.000411 |
| ABA |  |  |  |  |  |
| 17.1.1 | hormone metabolism.abscisic acid.synthesis-degradation | XLOC_011831 | AT1G16540 \| Symbols: SIR3 | 1.4505 | 0.000411 |
|  |  | XLOC_011057 | AT5G42560 \| Symbols: \| abscisic acid-responsive HVA22 family protein | 1.2172 | 0.00076 |
|  |  | XLOC_027197 | AT2G27150 \| Symbols: AAO3 | 1.1369 | 0.014871 |
|  |  | XLOC_011832 | AT1G16540 \| Symbols: SIR3 | 1.20741 | 0.002815 |
|  |  | XLOC_027196 | AT5G20960 \| Symbols: AAO1 | 1.02712 | 0.022431 |
| 17.1.2 | hormone metabolism.abscisic acid.signal transduction | XLOC_028331 | AT4G34000 \| Symbols: ABF3 | 2.82633 | 0.000411 |
|  |  | XLOC_010730 | AT3G19290 \| Symbols: ABF4 | 1.15303 | 0.002266 |
| 17.1.3 | hormone metabolism.abscisic acid.induced-regulated-responsive-activated | XLOC_015083 | AT1G74520 \| Symbols: ATHVA22A | 1.47613 | 0.000411 |
|  |  | XLOC_036063 | AT5G50720 \| Symbols: ATHVA22E | 2.7187 | 0.000411 |
|  |  | XLOC_025924 | AT5G13200 \| Symbols: \| GRAM domain-containing protein / ABA-responsive protein-related | -1.01992 | 0.002266 |
|  |  | XLOC_038600 | AT1G28200 \| Symbols: FIP1 | 1.56229 | 0.003346 |

**Supplementary Table 2.** The qRT-PCR validation values for the 25 selected genes.

| Gene name | Gene symbol | RNA-seq | qRT-PCR |
| --- | --- | --- | --- |
| class IV chitinase | XLOC_056159 | 3.34015 | 2.068 |
| NPR1 | XLOC_012174 | 0.930665 | 0.75675 |
| WRKY53 | XLOC_037606 | 2.44604 | 1.8745 |
| WRKY33 | XLOC_019616 | 2.15673 | 1.456 |
| WRKY70 | XLOC_019719 | 2.0157 | 0.66 |
| nrp | XLOC_018622 | 1.16842 | 0.77325 |
| CCCH | XLOC_024768 | 2.0425 | 1.4785 |
| RFL1 | XLOC_008440 | 3.26843 | 3.4095 |
| MPK3 | XLOC_027731 | 1.78 | 1.37125 |
| AEC | XLOC_003159 | -4.8897 | -4.72325 |
| ERF1 | XLOC_017562 | -1.72 | -1.65875 |
| NR1 | XLOC_010134 | -2.47317 | -3.08825 |
| DHS1 | XLOC_002181 | -3.56899 | -4.57775 |
| AAT2 | XLOC_001682 | -1.8467 | -2.19125 |
| ACLB2 | XLOC_031075 | -2.54381 | -3.003 |
| RPT5A | XLOC_009467 | -1.92444 | -1.48375 |
| CSB3 | XLOC_027636 | -1.92118 | -2.26625 |
| NAD | XLOC_023886 | -1.47378 | -1.41525 |
| EDA9 | XLOC_027598 | -2.917 | -3.0845 |
| OPR2 | XLOC_038293 | -5.14 | -5.455 |
| ABA3 | XLOC_011831 | 1.4505 | 1.412025 |
| CSLD3 | XLOC_008585 | 3.57897 | 3.834725 |
| XTR6 | XLOC_012121 | 2.75824 | 3.4369 |
| GH3.1 | XLOC_027203 | 1.96314 | 1.129475 |
| AAO3 | XLOC_027197 | 1.1369 | 1.184825 |

The third and fourth column were the log2(Fold Change) of the genes when compared blight to healthy samples from RNA-seq analysis and qRT-PCR analysis, respectively.

**Supplementary Table 3.** Primer sequences used to amplify the selected genes

| gene number | gene name | primer sequence (5'-3') | fragement size (in base pair) | Note |  |
| --- | --- | --- | --- | --- | --- |
| XLOC_012174 | NPR1 | TGTGCGGTAAAGCTTGTGAG | 105 | SA dependent, defense realted | |
|  |  | CAATGTGTTGTGGCAAGGTC |  |  |  |
| XLOC_026451 | PR1 | CTAGGGCACAGGTTGGTGTT | 137 | defense |  |
|  |  | ATGCAAGGTTCTCGCCATAC |  |  |  |
| XLOC_019616 | WRKY33 | CGATCCCACCTTCTGGTTTA | 179 | SA, negative regulater | |
|  |  | AGAAGGCCTTGTTTGGGTTT |  |  |  |
| XLOC_019719 | WRKY70 | CGCAACCAATCATCATCATC | 150 | SA positive regulater | |
|  |  | CAAGCTGTGAGCAGAAGCAG |  |  |  |
| XLOC_037606 | WRKY53 | AGCATTGGCTTCGACTTCAT | 109 | SA positive regulater | |
|  |  | TACCCCATTTGCTTCTTTCG |  |  |  |
| XLOC_056159 | chitinase IV | TGGCCCTGCTTAATGTTTTC | 142 | disease resistance | |
|  |  | CAAGTTGCTGGAGCCTGATT |  |  |  |
| XLOC_027731 | MPK3 | TTGAAGCCCAGCAATCTCTT | 188 | disease resistance | |
|  |  | CAGCCAACAGACCACACATC |  |  |  |
| XLOC_008440 | RFL1 | GTGACCGTGACAAGGTGTTG | 133 | disease resistance | |
|  |  | CTAACCCCGTGGTGTTGACT |  |  |  |
| XLOC_024768 | CCCH-type zinc finger gene | GCCTGTGGTTCTGATGGTTT | 190 | multiple stress induced | |
|  |  | TTGCCCTTTTTCTTGAATGG |  |  |  |
| XLOC_018622 | NRP | CAAAGCCAGGTCATTTTGGT | 131 | multiple stress induced | |
|  |  | GCCACTCTTGCTTCCAAGTC |  |  |  |
| XLOC_023886 | NAD | AGGAGCCATTTTTGCTGATG | 104 | malate dehydrogenase | |
|  |  | CGAAAAAGGGAAGCTCAGTG |  |  |  |
| XLOC_027636 | CSB3 | TACGGAACCACCAGAAAAGG | 137 | repressed by SA | |
|  |  | CAGAACGACGCTGAAAATCA |  |  |  |
| XLOC_003159 | AEC | CTTCGGAGCAGTCGTATGGT | 245 | auxin effux carrier | |
|  |  | CGGCAGCAGCTTTAACTACC |  |  |  |
| XLOC_012121 | XTR6 | TGGCAACTCTGCTGGTACTG | 177 | cell wall modification, IAA induced | |
|  |  | GGGTCAAACCAGAGGTGAAA |  |  |  |
| XLOC_011831 | ABA3 | GATGCTTTTGCAATCCTGGT | 90 | ABA synthesis regulator | |
|  |  | AGCAAACATGTCCAGCCTCT |  |  |  |
| XLOC_016830 | GAPC2 | GAAAGGTCTTGCCTGCTTTG | 103 | reference | |
|  |  | TCCTTCTCCAGCCTCACTGT |  |  |  |
| XLOC_017562 | ERF1 | ATCATTCTCGTGGGATGAGC | 111 | Ethylene-responsive transcription factor 1 | |
|  |  | GTTGTTGTCCCTTCGGCTAA |  |  |  |
| XLOC_010134 | NR1 | TTGAGGTTCTCGACCTGCTT | 180 | nitrate reductase | |
|  |  | GCTCAAACACGATTCCGATT |  |  |  |
| XLOC_002181 | DHS1 | AGCACAGTGAGCAGGGAGAT | 104 | Encodes a 2-deoxy-D-arabino-heptulosonate 7-phosphate (DAHP) synthase, which catalyzes the first committed step in aromatic amino acid biosynthesis | |
|  |  | CATGGCAGGATGATCAAGTG |  |  |  |
| XLOC_009467 | RPT5A | CTGGACACACTGCCTTCTGA | 133 | Encodes RPT5a (Regulatory Particle 5a), one of the six AAA-ATPases of the proteasome regulatory particle | |
|  |  | GCAGCACAATAGCCTCAACA |  |  |  |
| XLOC_001682 | AAT2 | CGTTAGGGCCTTGGTTGTAA | 203 | involved in Nitrogen metabolism | |
|  |  | TCTCACCATACCCCATGGAT |  |  |  |
| XLOC_031075 | ACLB2 | AATTATTGCCGAAGGTGTGC | 141 | encoding subunit B of the cytosolic enzyme ATP Citrate Lyase (ACL) | |
|  |  | GTTCCGGCTGTGTCACCTAT |  |  |  |
| XLOC_027598 | EDA9 | TGAAATTGCTGAGGCTGTTG | 176 | Encodes a 3-phosphoglycerate dehydrogenase that is essential for embryo development | |
|  |  | CGGTTTTCACACCACTTCCT |  |  |  |
| XLOC_008585 | CSLD3 | ATTGTAATACCGGGGGAAGC | 156 | cellulose synthase like D3 | |
|  |  | CCTAGAATTGCAACCGGGTA |  |  |  |
| XLOC_027203 | GH3.1 | TTCTCGACTGCTCCGAGAAT | 155 | indole-3-acetic acid-amido synthetase | |
|  |  | AGCAGCACTGGTTCAGGACT |  |  |  |
| XLOC_027197 | AAO3 | GGTGAGCAGGAGCAGGATAG | 90 | aldehyde oxidase delta isoform catalyzing the final step in abscisic acid biosynthesis | |
|  |  | AGAAGGGTCAACGCTTGAGA |  |  |  |
| XLOC_038293 | OPR2 | AATGGTGGTTTTCTCGTTGC | 105 | catalyze the final step of JA synthesis | |
|  |  | TTTCCAAGCTTCCACTTGCT |  |  |  |

**Supplementary Table 4.** The NCBI accession no. for DNA and RNA reads as well as the draft assembly

| Accession No. | Sample | note |
| --- | --- | --- |
| SRX502932 | 14_14 | DNA reads |
| SRX378262 | 14_14 | RNA reads |
| SRX504183 | 16_11 | DNA reads |
| SRX378263 | 16_11 | RNA reads |
| SRX504260 | 18_7 | DNA reads |
| SRX378264 | 18_7 | RNA reads |
| SRX504480 | 20_2 | DNA reads |
| SRX378265 | 20_2 | RNA reads |
| SRX505103 | 20_6 | DNA reads |
| SRX378266 | 20_6 | RNA reads |
| SRX374184 | 23_11 | DNA reads |
| SRX378269 | 23_11 | RNA reads |
| SRX505104 | 24_8 | DNA reads |
| SRX378270 | 24_8 | RNA reads |
| AZHM00000000 | 23_11 | Swingle citrumelo assembly |

**Supplementary notes.**

1. **Assembly of the Swingle citrumelo genome**

The 81,496,678×2 paired-end DNA reads from tree 23_11 were used for genome assembly and annotation. These raw reads were trimmed using CLC genomic workbench (V6.0.1, CLC Bio) and the following parameters: minimum quality score 0.05, maximum number of ambiguities 2 were used to trim the low quality reads; the reads containing adapters and reads shorter than 55 bp were discarded. The 69,656,379×2 trimmed paired-end reads with average length 97.6 bp were assembled using CLC genomic workbench (V6.0.1, CLC Bio) at a range of word size (24 (parameter: auto word size), 33, 39, 45). The contigs generated by assembly of word size 33 were chosen for further analysis because word size of 33 produced the longest (on average) contigs and highest matched reads (Table S5).

Table S5. Overview of assemblies of Swingle citrumelo reads using differential word sizes

|  | **Word sizes** | | | |
| --- | --- | --- | --- | --- |
|  | 24 | 33 | 39 | 45 |
| % reads matched | 68.3 | 69.2 | 68.6 | 67.8 |
| # contigs (×1000) | 739 | 720 | 710 | 707 |
| Average contig length (bp) | 662 | 669 | 660 | 646 |
| Assembly length (Mb) | 489 | 482 | 469 | 457 |

The contigs with average coverage higher than 6 were extracted, and then ordered and oriented against the sweet orange (*Citrus sinensis*) genome [3] using ABACAS (Algorithm Based Automatic Contiguation of Assembled Sequences) software [4]. The mapped contigs were anchored in the 9 chromosomes and the Unchr superscaffold of sweet orange genome and 10 pseudo-superscaffolds were formed, the pseudo-superscaffolds were broken to individual scaffolds if the newly formed gaps were longer than 500 bp. Those unmapped contigs were searched against *Citrus clementina* draft genome v0.9 downloaded from citrus genome database (<http://www.citrusgenomedb.org/species/clementina/genome0.9>) [1] and NCBI nr database using blastn with e-value cutoff of 1e-10. Finally, the scaffolds and contigs belonging to Citrus were pulled together and scaffolded using SSPACE [5]. The number of Ns in the assembly was reduced by filling the gaps in scaffolds using GapFiller [6].

1. **Assessment of the draft assembly**

The coding region coverage of the draft assembly was validated using the Core Eukaryotic Genes Mapping Approach (CEGMA) [7]. The accuracy of assembly was also assessed by aligning 7,954 available Swingle citrumelo ESTs downloaded from dbEST [8] to the draft assembly using sim4db [9] and exonerate [10].

1. **Genome annotation**

The draft genome annotation was created using MAKER2 pipeline [11]. The RNA-seq reads were mapped to the draft assembly using Tophat2 (ver. 2.0.7) [12] and the mapped paired-end reads were assembled using Trinity [13]. The assembled contigs together with cDNA sequences of *Citrus sinensis* and *Citrus clementina* genome were fed to MAKER2 as ESTs and alt-ESTs for the annotation, respectively. Repetitive elements were identified using RepeatMasker (version open-4.0.1) with Repbase repeat library ver. 20120418 [14] and the Carrizo repeats [15] download from <http://citrus.pw.usda.gov/> as well as using MAKER2 internal RepeatRunner package with its default repeat protein database [16]. SNAP, AUGUSTUS and GeneMark were employed for gene predictions within the MAKER pipeline [17-19]. Those MAKER models supported by EST and alt-EST evidences were kept in the annotation set; the *ab initio* predictions were scanned for protein domains using InterProScan [20] and those showed positive results were added to the annotation set. 44.8 Mb (16.8% of 280.6 Mb) of repetitive elements were identified in the draft assembly using RepeatMasker, generating a 235.8 Mb repeat-masked assembly for gene prediction. Following two cycles of MAKER run, 29,054 genes were predicted without detection of alternative splicing forms. The 29,054 genes were aligned to the custom protein database composing of 44,275 *Citrus sinensis* proteins from Xu et al (2013), 33,929 *Citrus clementina* proteins downloaded from citrus genome database (<http://www.citrusgenomedb.org/species/clementina/genome0.9>) [1] and 33,643 *Viridiplantae* (green plant, downloaded on 1^st^, April, 2013) proteins from Swiss-Prot database using blastp (e-value, 1e-5), the result demonstrated that 14,219 (48.9%) showed best hit to *Citrus sinensis* proteins, 13,719 (47.2%) showed best hit to *Citrus clementina* proteins, 186 (0.6%) showed best hit to *Viridiplantae* (green plant) proteins and 930 (3.2%) did not find hits in the database when cutoff e-value 1e-5 was applied.

1. **Phylogenetic relationship determination between Swingle citrumelo and other sequenced citrus cultivars**

The Illumina paired-end DNA reads from the eight sequenced citrus cultivars [1] were downloaded from NCBI SRA database. The reads from each sample as well as from the seven samples used in study were aligned to the sweet orange genome [3] using Bowtie2 (Ver. 2.0.6) [21] with –fast parameter. The generated alignment files were converted from sam to bam files using Samtools ver. 0.1.19+ [22]. The bam files were sorted and then quality filtered (option -bq 20) to remove reads whose alignment with MapQ smaller than 20. The SNPs were called from the filtered alignment bam files using mpileup and bcftools scripts integrated in Samtools. The generated “.vcf” file further filtered using vcftools ver. 0.1.13 with parameter --minGQ 20 (Danecek et al., 2011). Then the vcf file was fed to SNPhylo pipeline to construct the phylogenetic tree [2]. 100 boostrap replicates were made.

The DNA reads from the seven samples were also mapped to Swingle citrumelo assembly, SNPs were called using the same process described above. The SNPs were originated from the heterozygous sites in the Swingle citrumelo genome. We checked the first 10,000 SNPs sites manually and found 9,995 sites were identical for all the seven samples, thus we reasoned the seven samples were from nucellar seedlings

**References**

1. Wu GA, Prochnik S, Jenkins J, Salse J, Hellsten U, Murat F, Perrier X, Ruiz M, Scalabrin S, Terol J *et al*: **Sequencing of diverse mandarin, pummelo and orange genomes reveals complex history of admixture during citrus domestication**. *Nat Biotechnol* 2014, **32**(7):656-+.

2. Lee TH, Guo H, Wang X, Kim C, Paterson AH: **SNPhylo: a pipeline to construct a phylogenetic tree from huge SNP data**. *BMC genomics* 2014, **15**:162.

3. Xu Q, Chen L-L, Ruan X, Chen D, Zhu A, Chen C, Bertrand D, Jiao W-B, Hao B-H, Lyon MP *et al*: **The draft genome of sweet orange (Citrus sinensis)**. *Nature genetics* 2013, **45**(1):59-66.

4. Assefa S, Keane TM, Otto TD, Newbold C, Berriman M: **ABACAS: algorithm-based automatic contiguation of assembled sequences**. *Bioinformatics* 2009, **25**(15):1968-1969.

5. Boetzer M, Henkel CV, Jansen HJ, Butler D, Pirovano W: **Scaffolding pre-assembled contigs using SSPACE**. *Bioinformatics* 2011, **27**(4):578-579.

6. Boetzer M, Pirovano W: **Toward almost closed genomes with GapFiller**. *Genome Biol* 2012, **13**(6).

7. Parra G, Bradnam K, Korf I: **CEGMA: a pipeline to accurately annotate core genes in eukaryotic genornes**. *Bioinformatics* 2007, **23**(9):1061-1067.

8. Boguski MS, Lowe TMJ, Tolstoshev CM: **Dbest - Database for Expressed Sequence Tags**. *Nature genetics* 1993, **4**(4):332-333.

9. Walenz B, Florea L: **Sim4db and Leaff: utilities for fast batch spliced alignment and sequence indexing**. *Bioinformatics* 2011, **27**(13):1869-1870.

10. Slater GS, Birney E: **Automated generation of heuristics for biological sequence comparison**. *Bmc Bioinformatics* 2005, **6**.

11. Holt C, Yandell M: **MAKER2: an annotation pipeline and genome-database management tool for second-generation genome projects**. *BMC Bioinformatics* 2011, **12**.

12. Kim D, Pertea G, Trapnell C, Pimentel H, Kelley R, Salzberg S: **TopHat2: accurate alignment of transcriptomes in the presence of insertions, deletions and gene fusions**. *Genome Biol* 2013, **14**(4):R36.

13. Haas BJ, Papanicolaou A, Yassour M, Grabherr M, Blood PD, Bowden J, Couger MB, Eccles D, Li B, Lieber M *et al*: **De novo transcript sequence reconstruction from RNA-seq using the Trinity platform for reference generation and analysis**. *Nat Protoc* 2013, **8**(8):1494-1512.

14. Jurka J, Kapitonov VV, Pavlicek A, Klonowski P, Kohany O, Walichiewicz J: **Repbase update, a database of eukaryotic repetitive elements**. *Cytogenetic and Genome Research* 2005, **110**(1-4):462-467.

15. Belknap WR, Wang Y, Huo N, Wu J, Rockhold DR, Gu YQ, Stover E: **Characterizing the citrus cultivar Carrizo genome through 454 shotgun sequencing**. *Genome* 2011, **54**(12):1005-1015.

16. Smith CD, Edgar RC, Yandell MD, Smith DR, Celniker SE, Myers EW, Karpen GH: **Improved repeat identification and masking in Dipterans**. *Gene* 2007, **389**(1):1-9.

17. Korf I: **Gene finding in novel genomes**. *Bmc Bioinformatics* 2004, **5**.

18. Stanke M, Keller O, Gunduz I, Hayes A, Waack S, Morgenstern B: **AUGUSTUS: *ab initio* prediction of alternative transcripts**. *Nucleic Acids Res* 2006, **34**:W435-W439.

19. Lomsadze A, Ter-Hovhannisyan V, Chernoff YO, Borodovsky M: **Gene identification in novel eukaryotic genomes by self-training algorithm**. *Nucleic Acids Res* 2005, **33**(20):6494-6506.

20. Zdobnov EM, Apweiler R: **InterProScan - an integration platform for the signature-recognition methods in InterPro**. *Bioinformatics* 2001, **17**(9):847-848.

21. Langmead B, Salzberg SL: **Fast gapped-read alignment with Bowtie 2**. *Nat Methods* 2012, **9**(4):357-U354.

22. Li H, Handsaker B, Wysoker A, Fennell T, Ruan J, Homer N, Marth G, Abecasis G, Durbin R, Proc GPD: **The Sequence Alignment/Map format and SAMtools**. *Bioinformatics* 2009, **25**(16):2078-2079.
